# A Grape-Derived Solvent for the Recovery of Phenolic Compounds from Food Waste By-Products Using Ultrasonic-Assisted and Overnight Extraction

**DOI:** 10.3390/molecules30193878

**Published:** 2025-09-25

**Authors:** Dimitrios G. Lazaridis, Vassilios K. Karabagias, Nikolaos D. Andritsos, Aris E. Giannakas, Ioannis K. Karabagias

**Affiliations:** Department of Food Science and Technology, School of Agricultural Sciences, University of Patras, G. Seferi 2, 30100 Agrinio, Greece; up1076474@ac.upatras.gr (D.G.L.); nandritsos@upatras.gr (N.D.A.);

**Keywords:** red onions, grape pomace, food waste, polyphenols, extraction, solvents, eco-friendly, Merlot, Syrah

## Abstract

This present study aimed to investigate the recovery of polyphenols from red onion peel (OPP) and pomace of Merlot (MWP) and Syrah (SWP) grape varieties, using a common biphasic solvent (70/30 ethanol/water) and a new biphasic and eco-friendly solvent that has been developed in our laboratory (ethanol of grape origin). Moreover, overnight and ultrasonic-assisted extractions were carried out to investigate whether the extraction method could affect the obtained results. Results showed that 70% ethanol achieved a significantly (*p* < 0.05) higher yield in total phenolic content (TPC) and total flavonoid content (TFC), while the extracts with ethanol of grape origin exhibited considerably higher antioxidant activity as evidenced by the DPPH and complementary by FRAP assays. The overnight and ultrasonic-assisted extraction played a significant (*p* < 0.05) role in achieving better extraction of bioactive components such as phenolic compounds. Color parameters were also determined, showing that the presence of yellow, blue and red color tones depend on the extraction method and solvent, due to the different compositional characteristics of pigments, mainly anthocyanins. In addition, physicochemical parameters such as pH and total soluble solids (TSSs) of the extracts were also measured. Finally, the composition of ethanol of grape origin was characterized by means of Ultraviolet–Visible (UV-Vis) and Attenuated Total Reflectance-Fourier Transform Infrared (ATR-FTIR) spectroscopy, which confirmed the presence of ethanol and acetic acid. This study brings new results regarding the prospect of using new solvents for the recovery of bioactive compounds from agricultural by-products, and the development direction of scientific research or industrial production, based on ultrasonic-assisted and overnight extraction methods.

## 1. Introduction

Food waste is a global problem that has serious economic, social, and environmental implications [1]. In recent years, this problem has grown exponentially, as it has been reported that more than 50% of various plant products, including fruit and vegetables, are being wasted after post-harvesting processes [2,3]. These plant-derived by-products and wastes are mainly peels, seeds, pomace, leaves, stalks, and skins, and can be obtained from a variety of fruits and vegetables [4]. Moreover, it is crucial to note that these by-products are rich sources of bioactive phytochemicals, such as proteins, polysaccharides, essential oils, organic acids, lipids, and polyphenols, that in turn can be valorized and exploited [5]. Tapia-Quiros et al. [6] and Castro-Muñoz et al. [7] stated that the recovery of polyphenols from waste and their applications in the cosmetic, pharmaceutical, and food industries are really interesting. Polyphenols are secondary plant metabolites of high added value, associated with antibacterial, anti-inflammatory, and anti-carcinogenic effects [6,7,8]. They consist of at least one phenol group per molecule, while they have other functional groups in their structure, such as carboxyl and multiple hydroxyl groups. The presence of these groups in the phenolic structure makes it a strong hydrogen (H^−^) donor, neutralizing free oxygen radicals and acting as an antioxidant compound [9].

One of the most consumable foods around the world is the onion (*Allium cepa* L.). It has been a domesticated plant for over 4000 years, as evidenced by an Egyptian image from 3000 BC and a Sumerian written paper from 2600 to 2100 BC [10]. In 2019, 200 million tons of shallots and onions were produced worldwide, making onions one of the most cultivated plants globally [11]. Their overall production is estimated at 84.573 million metric tons (MMT), with China and India contributing to 67.5% of the global production [12]. Onions are cultivated for their multilayer edible bulb that can be consumed in various forms (fresh, baked, boiled, pickled, etc.), while they offer multiple health benefits (anti-inflammatory, anti-cancer, and hepatoprotective effects) to human nutrition [10,13,14]. However, a considerable amount of food waste occurs from the non-edible parts of the onion, including the top and bottom parts, outer skins and peels, and the first two outer layers [13,15]. Their increased production in recent years generates increased waste of skins, peels, roots, flowers, and damaged bulbs [12]. Only in India, the onion waste reaches 500 kg every day, including peels and skin [16]. The occurring waste can have multiple environmental effects if not properly disposed of, whereas it cannot be used for fodder preparation or fertilizer, due to the strong onion aroma that is released [13,17]. Therefore, other practices of utilization should be found. Onion peels are rich in bioactive compounds such as polyphenols, flavonoids, anthocyanins, and tannins, which can be valorized from the pharmaceutical, biomedicine, or food industry sectors [13,17,18,19]. Moreover, they consist of important proteins, minerals, dietary fibers, and polysaccharides [13]. Furthermore, it has been documented that onion peels contain flavonols, and the majority of these polyphenols are glucose derivatives of kaempferol and quercetin [18,20], while more than 80% of the total flavonoid content consists of quercetin derivatives [18]. The high concentration of quercetin, mostly in the outer layers of onions, is mainly attributed to the sunlight exposure of bulbs [21,22].

On the other hand, a horticultural crop that plays an important role in the economy of many countries around the Mediterranean zone is grapes (*Vitis vinifera* L.) [2], while China is the global leader in grape production [23]. According to USDA [24], raw grapes consist of 80.54% water and 15.48% sugars. After their harvest, red grapes are crushed and their juice, with all the solids that grapes consist of (peels, seeds, stalks), proceeds to the fermentation stage, where the total sugars are consumed by *Saccharomyces cerevisiae*, producing ethanol through biochemical processes [2]. After the wine collection, what is left is a mass of stalks, seeds, and skins, which is called grape pomace. Grape pomace has been used as animal feed or fertilizer in the Mediterranean countries [25], even though it contains a high amount of bioactive compounds with antibacterial, anti-inflammatory, and antioxidant properties [26]. These compounds are mainly stilbenes, flavonoids, phenolic acids, tannins, and anthocyanins [27]. Numerous studies in the literature extracted several bioactive compounds (gallic acid, epicatechin, epigallocatechin, syringic acid, and quercetin) with different methods and solvents and determined their concentration [28,29,30,31,32]. Verano-Naranjo et al. [33] developed ethanolic and hydroethanolic extracts of grape pomace and identified several phenolic compounds (i.e., flavonols, flavanols, and anthocyanins) by Ultra-High-Performance Liquid Chromatography–Electrospray Ionization–Time of Flight–Mass Spectrometry (UHPLC-ESI-ToF-MS). Finally, these extracts exhibited antibacterial activity against *Staphylococcus aureus*. Overall, the identification and recovery of bioactive compounds from grape pomace is a topic that encompasses a wide research field.

Based on the above and in parallel considering the gap in the literature, mainly in the recovery of phenolic compounds from red onion waste using emerging green solvents (i.e., ethanol of grape origin), the scope of the present work was to compare different solvents and extraction methods, aiming for the best recovery of total phenolic compounds from agro-industrial by-products such as red onion peel powder and winery pomace of Merlot and Syrah grape varieties. A new solvent that was applied in our laboratory (ethanol of grape origin) was compared with ethanol (70% ethanol–30% water), both subjected to two different extraction methods (overnight and ultrasonic-assisted extraction). The study contributes to the development and valorization of novel and green methods for the recovery of phenolic compounds from food waste by-products, which could soon be utilized for potential food [2] or pharmaceutical applications.

## 2. Results and Discussion

### 2.1. Recovery of Phenolic Compounds

The total phenolic content (TPC) of the OPP, MWP, and SWP extracts subjected to different solvents and extraction methods is represented in Table 1. Concerning the OPP extracts, their total phenolic content ranged between 15.58 ± 0.43 and 16.59 ± 0.96 mg GAE/g, with no statistically significant (*p* > 0.05) differences between the values, indicating that the solvent and extraction method did not affect the recovery of phenolic compounds in OPP. Kim et al. [34] studied two different red onion cultivars harvested in Korea and extracted in an 80% methanol/water solution. The respective content of total phenolic compounds was 1506.22 ± 138.08 and 2035.56 ± 16.67 mg GAE/100 g of fresh weight. Similarly, Nil et al. [35] reported that 80% aqueous methanol and 80% aqueous ethanol achieved the greatest yield of total phenolic compounds (415.3 ± 6.2 and 398.5 ± 5.4 mg GAE/g of dry extract, respectively) from red onion skins, against different solvents (ethyl acetate: 305.9 ± 1.5 mg GAE/g, diethyl ether: 92.6 ± 1.2 mg GAE/g, butanol: 115.6 ± 2.1 mg GAE/g, and water: 30.5 ± 1.3 mg GAE/g). These findings highlight the importance of biphasic solvents in the extraction of phenolic compounds.

On the other hand, in the case of MWP, the different solvents significantly (*p* < 0.05) affected the content of phenolic compounds. Specifically, the results of overnight extraction (9.43 ± 0.19 mg GAE/g) and ultrasonic-assisted extraction (8.61 ± 0.12 mg GAE/g) with ethanol of grape origin were not significantly (*p* > 0.05) different. In the case of the 70% ethanol, the ultrasonically assisted extract had a statistically significant (*p* < 0.05) highest recovery of phenolic compounds (16.40 ± 0.93 mg GAE/g), compared to the 70% aqueous ethanol extract subjected to overnight extraction (11.62 ± 1.91 mg GAE/g). Similarly, 70% ethanol was the most effective solvent for the extraction of phenolic compounds from SWP, using ultrasonic extraction (15.68 ± 0.96 mg GAE/g) and the overnight extraction method (14.43 ± 1.46 mg GAE/g). The solvent played a statistically significant (*p* < 0.05) role in their total recovery compared to extracts of ethanol of grape origin, using overnight extraction (11.12 ± 0.20 mg GAE/g) and ultrasonic-assisted extraction (9.93 ± 0.26 mg GAE/g) methods. Recently, Kadouh et al. [32] compared six red wine grape pomace extracts in 50% aqueous acetone, reporting that Merlot and Syrah extracts had the highest total phenolic content (0.29 and 0.28 mg GAE/mg, respectively). Moreover, Martins et al. [36] reported that enzymes release the phenolic acids from grape pomace, increasing the total phenolic content. Considering the above, it is evident that different treatments and extraction methods on grape pomace primarily affect the concentration of phenolic compounds, as we also tried to highlight in the present study using pomace of Greek red grape cultivars.

Before going any further in the discussion of the findings of this research, it is important to comment on how the findings and the hypothesis of the present study advance the field beyond existing literature regarding the use of emerging green solvents such as deep eutectic solvents (i.e., urea, glycerol, ethylene glycol, etc.) or subcritical water. To the best of our knowledge, there is no published study in the literature reporting the recovery of phenolic compounds efficiently from food waste byproducts using ethanol of grape origin. The use of deep eutectic solvents has been previously used for the isolation of polysaccharides, proteins, DNA, or RNA. However, their high viscosity, cost, and questionable safety limit their use as extraction solvents [37]. Regarding the use of subcritical water, it has been applied efficiently for the recovery of phenolic compounds from plant extracts, providing better recovery results compared to conventional organic solvents (i.e., ethanol, methanol). However, the most important parameter when using subcritical water as an extraction solvent is the choice of the temperature at which the extraction is performed. Normally, the total yield of the extraction increases with increasing temperature [38]. However, in cases where low-to-moderate temperatures are applied for the extraction of phenolic compounds from plant-derived food waste by-products (such as in our case), subcritical water may not be an ideal solvent.

### 2.2. Total Flavonoid Content

The total flavonoid content of OPP, MWP, and SWP extracts is represented in Table 1. Overall, 70% ethanol turned out to be a better solvent for the extraction of flavonoids from all the powder samples. Furthermore, ultrasonic extraction was the optimal method using ethanol of grape origin, while overnight extraction turned out to be better using 70% ethanol. These results indicate that there are different optimal extraction methods for each solvent. This finding has also been confirmed previously by several studies, where researchers noticed that the presence of ethanol achieves a higher concentration of flavonoids in the extracts [33,39]. Specifically, in the case of OPP, extracts with ethanol of grape origin showed no statistically significant (*p* > 0.05) differences, compared to overnight (2.01 ± 0.02 mg QE/g) and ultrasonic-assisted (2.23 ± 0.28 mg QE/g) extraction. On the other hand, OPP extracts obtained with 70% ethanol had significantly (*p* < 0.05) different total flavonoid content. Similar results were reported in the study of Lee et al. [39], where researchers compared ethanolic, hot water, and subcritical water extracts. Moreover, Oancea et al. [19] studied 70% ethanolic extracts from red onion skins using ultrasonic-assisted extraction with various solvent/solid ratios and extraction times. The total flavonoid concentration (121.49 ± 27.59 mg QE/g dry matter) of the optimal conditions used was higher compared to the results of the present study.

As previously mentioned, 70% ethanol turned out to be the best solvent for the extraction of flavonoids. This also applies in the case of extracts from grape pomace. The total flavonoid content of MWP extracts obtained with ethanol of grape origin using overnight (1.91 ± 0.06 mg QE/g) and ultrasonic-assisted (1.99 ± 0.36 mg QE/g) extraction was significantly (*p* < 0.05) different compared with that of the 70% ethanolic extracts using ultrasonic-assisted (5.52 ± 0.27 mg QE/g) and overnight (5.63 ± 0.03 mg QE/g) extraction methods. In the same line of reasoning, the total flavonoid content of SWP obtained with 70% ethanol and subjected to overnight extraction was significantly (*p* < 0.05) higher (6.28 ± 0.62 mg QE/g) compared to the other treatments (Table 1). Numerous studies in the literature [28,29,32,40,41] have confirmed the presence of flavonoids in red grape pomace extracts, supporting the results of the present study.

### 2.3. Total Anthocyanin Content

The total anthocyanin content of OPP, MWP, and SWP EEGO extracts is represented in Table 1. Unfortunately, there is no data for the 70% ethanolic extracts, as the solution became blurry and cloudy after the reaction, making the measurements impossible. This is probably owed to the instability and degradation of anthocyanins in ethanol [42]. Also, Taghavi et al. [43] reported that the anthocyanins are extracted better in acidified solvents with low pH. In our study, the pH of ethanol of grape origin was 2.16, while the pH of 70% ethanol was 7.20.

Overnight extraction yielded a higher total anthocyanin content in OPP extracts (0.10 ± 0.00 mg C3G/g) compared to extracts treated with ultrasonication (0.08 ± 0.00 mg C3G/g). In contrast, ultrasonic-assisted extraction resulted in a higher total anthocyanin content for MWP and SWP. MWP extracts recorded the highest total anthocyanin content using ultrasonic-assisted extraction (0.62 ± 0.00 mg C3G/g), followed by overnight extraction (0.45 ± 0.00 mg C3G/g). Finally, the SWP extracts had similar values when subjected to ultrasonic-assisted and overnight extraction (0.28 ± 0.00 mg C3G/g in both cases). As we mentioned before, red onion peels and grape pomace are rich sources of anthocyanins. Specifically, numerous research studies reported similar results when investigating the total anthocyanin content of red onion peels [18,19,34,44], while the main anthocyanin is cyanidin 3-glucoside [45,46], among others, as we mentioned above. Similarly, additional studies in the literature have reported on the total anthocyanin content of different grape varieties [29,30] using ultrasonic-assisted extraction [47]. The most determined anthocyanins of red grape pomace are mainly cyanidin, malvidin, petunidin, and delphinidin glucosides [48].

### 2.4. Antioxidant Activity

The antioxidant activity of all treated samples is shown in Table 2. In contrast with the previous findings, the antioxidant activity of all extracts obtained with ethanol of grape origin was significantly (*p* < 0.05) higher with respect to the ultrasonically assisted extracts. The OPP extract obtained with ethanol of grape origin after overnight extraction inhibited the DPPH by 63.70 ± 1.43%, followed by that treated with ultrasonic-assisted extraction (62.60 ± 0.69%), showing no statistically significant (*p* > 0.05) differences. Similarly, the 70% ethanolic extracts had no statistically significant (*p* > 0.05) differences, recording values of 53.70 ± 2.18% and 54.82 ± 1.38% concerning the DPPH^.^ inhibition in the case of overnight and ultrasonic-assisted extraction, respectively. Similar results were reported in the works of Lee et al. [39] and Oancea et al. [19], who studied the antioxidant activity of orange and red skin onion peels, respectively, against the DPPH^.^ free radical.

The extract of MWP obtained with ethanol of grape origin and subjected to overnight extraction exhibited the highest antioxidant activity (71.17 ± 0.34%), followed by the ultrasonic-assisted extract (67.86 ± 1.73%), with statistically significant (*p* < 0.05) differences. Furthermore, the ultrasonically assisted and overnight extracted samples of MWP with 70% ethanol recorded antioxidant activity values of 53.29 ± 0.94% and 52.94 ± 1.66, respectively. On the same path, the extracts of SWP obtained with ethanol of grape origin recorded nearly the same values in the case of overnight (68.82 ± 2.55%) and ultrasonic-assisted extraction (68.85 ± 2.40%), followed by those treated with 70% ethanol during overnight (50.56 ± 1.50%) and ultrasonic-assisted (46.23 ± 3.39%) extraction. The antioxidant activity of grape pomace and red grape skins was reported in previous studies [40,49,50,51]. Present results indicate that the extraction method was efficient only in the case of using ethanol of grape origin for the extraction of MWP, while in the rest of the treatments, it was noticed that the extraction method did not play a significant role (in relation to the solvent) in antioxidant activity values, allowing us to select the cheapest and eco-friendly solvent (ethanol of grape origin).

The aforementioned pattern was also highlighted in a previous study, where the hot-water extracts of different plant products and wastes resulted in higher antioxidant activity than the ethanolic ones. However, the TPC was higher in the ethanolic extracts. The authors supported that the extracted compounds differ with respect to the extraction methods, while it is important to find the best protocol for the efficient extraction of phenolic compounds [52]. In our opinion, due to its higher water content than 70% ethanol, ethanol of grape origin extracted more water-soluble compounds that react with the DPPH. (i.e., ascorbic acid) [34,53,54], tocopherol [34,55,56], cysteine and glutathione [57,58,59,60], thus resulting in the higher antioxidant activity of the extracts obtained with ethanol of grape origin, even though 70% ethanol extracted more efficiently the phenolic compounds. Apart from the expression of antioxidant activity values in percentage, we estimated the values in Trolox equivalents (and mmol Fe^2+^/L) using the ferric-reducing antioxidant power (FRAP) assay, during which important findings were obtained for the antioxidant activity of OPP, MWP, and SWP extracts concerning overnight and ultrasonic-assisted extraction. In the case of OPP, the highest FRAP values (1149.65 ± 17.06 μmol TE/L) were recorded by using 70% ethanol in overnight extraction, and the lowest (1102.59 ± 8.23 μmol TE/L) was recorded in the case of using ethanol of grape origin during overnight extraction. Similarly, in MWP, the highest FRAP values (1189.94 ± 10.29 μmol TE/L) were recorded by using 70% ethanol in ultrasonic-assisted extraction, and the lowest (1076.12 ± 14.12 μmol TE/L) was recorded in the case of using ethanol of grape origin during overnight extraction. Finally, in SWP (as in the case of MWP), the highest FRAP values (1249.06 ± 19.41 μmol TE/L) were recorded by using 70% ethanol in ultrasonic-assisted extraction, and the lowest (1113.76 ± 9.41 μmol TE/L) was recorded in the case of using ethanol of grape origin during overnight extraction. In most cases, ultrasonic-assisted extraction resulted in a better recovery of phenolic compounds with antioxidant activity, as shown also by the FRAP assay. Even though slight differences were recorded by using ethanol of grape origin and 70% ethanol for the estimation of the antioxidant activity of all extracts between the DPPH and FRAP assays during overnight or ultrasonic-assisted extraction (Table 2), the present findings allow us to insist again on the use of a cheaper and more friendly solvent for the extraction of bioactive compounds from food waste by-products.

### 2.5. Color Parameters

#### 2.5.1. Yellow Color Tone

The color parameters of the studied extracts are represented in Table 3. The color is an important indicator of the freshness of fruits and vegetables [61]. In our case, the color of the food waste extracts plays a significant role in their further utilization and different applications. The OPP extracts recorded the highest %YCT, ranging between 79.78 ± 0.09% for the extracts obtained with ethanol of grape origin and ultrasonically treated, and 76.25 ± 0.08% for those obtained with 70% ethanol during ultrasonic-assisted extraction, with statistically significant (*p* < 0.05) differences. Quite close values were recorded for the overnight extracted samples with 70% ethanol (78.55 ± 0.07%) and ethanol of grape origin (77.99 ± 0.26%). On the other hand, MWP and SWP extracts recorded lower %YCT values. Briefly, the MWP extracts using 70% ethanol exhibited no statistically significant (*p* > 0.05) different values, concerning the extraction method (overnight: 45.72 ± 0.16% and UAE: 45.84 ± 0.38%), in contrast with those treated with ethanol of grape origin overnight (37.43 ± 0.02%) and with ultra sonication (39.94 ± 0.01%), which differed significantly (*p* < 0.05). Finally, the SWP extracts recorded statistically significant (*p* < 0.05) different values among the treatments. The lower %YCT values were obtained in the case of using ethanol of grape origin overnight (42.57 ± 0.09%), followed by those treated with ultrasonication (43.65 ± 0.10%), 70% ethanol with ultrasonic-assisted extraction (51.90 ± 0.24%), and 70% ethanol overnight (53.22 ± 0.08%). We noticed that in the case of grape pomace (MWP and SWP), 70% ethanol achieved higher values. This finding is owed to the fact that the color of red grapes (and, as a consequence, that of grape pomace) depends on the presence of pigments (i.e., anthocyanins) [62], which are extracted in higher concentration using a specific solvent (see Section 2.1). Furthermore, the yellow color of the extracts is due to the presence of carotenoids [63] and anthoxanthins, which are types of flavonoid pigments, including major flavonoids such as quercetin, myricetin, and luteolin [64]. These compounds are responsible for this white to creamy yellow color. In our opinion, the high quercetin content of OPP [18] is the main reason that the OPP extracts recorded the highest %YCT values, compared to those of MWP and SWP extracts. Finally, pH plays a significant role in the stability of pigments [65], as anthoxanthins develop a more yellowish color in more alkaline pH (see next sections).

#### 2.5.2. Blue Color Tone

The blue color tone of the treatments is shown in Table 3. Concerning the OPP extracts, the %BCT values were significantly (*p* < 0.05) different, varying from 17.03 to 19.13%. The highest value at MWP and SWP extracts obtained by EEGO overnight (48.18 ± 0.01% and 45.57 ± 0.11%, respectively), followed by EEGO UAE (43.13 ± 0.01% and 38.17 ± 0.11, respectively), while the MWP extracts with 70% aqueous ethanol subjected to overnight and ultrasonic assisted extraction had almost similar values (40.13 ± 0.17% and 40.00 ± 0.36%, respectively). In contrast, the SWP extracts with 70% aqueous ethanol showed statistically significant (*p* < 0.05) differences, concerning the overnight (35.68 ± 0.07%) and ultrasonic-assisted (34.99 ± 0.22%) extraction. The higher %BCT of grape pomace (MWP and SWP) EEGO extracts is mainly attributed to the water-soluble red to blue color anthocyanins [63]. Moreover, the number of hydroxyl groups in the B-ring plays an important role, because the blue color deepens as their number increases [66]. Furthermore, the presence of delphinidin in grape skins [67,68], an anthocyanidin that is responsible for the red-blue color in grapes [69,70], strengthens the blue color of grape pomace extracts, especially when they are in a pH range between 3.2 and 5.2 (see next sessions) [71]. Myrtillin (delphinidin-3-O-glucoside) has also been found in the grape pomace of several grape varieties [72].

#### 2.5.3. Red Color Tone

Table 3 represents the %RCT values of OPP, MWP, and SWP extracts using different solvents and extraction methods. The lowest %RCT values among the powders were recorded in the OPP extracts that exhibited statistically significant (*p* < 0.05) differences between the treatments. Ultrasonic-assisted extraction with 70% ethanol achieved the highest values (5.10 ± 0.04%), followed by those of using overnight ethanol of grape origin (4.19 ± 0.07%), 70% ethanol overnight (4.00 ± 0.07%), and ultrasonic-assisted extraction with ethanol of grape origin (3.09 ± 0.04%). In the case of MWP, ultrasonic-assisted extraction with ethanol of grape origin achieved the highest yield among MWP extracts (16.92 ± 0.01%), followed by those using ethanol of grape origin overnight (14.39 ± 0.02%) with statistically significant (*p* < 0.05) differences. The 70% ethanol recorded the lower values, while the extraction method did not have a statistically significant (*p* > 0.05) effect [overnight (14.14 ± 0.11%) and ultrasonic-assisted (14.16 ± 0.18%) extraction]. On the other hand, SWP extracts recorded statistically significant (*p* < 0.05) differences with respect to the solvent and extraction methods. Briefly, ethanol of grape origin and 70% ethanol were more efficient in the extraction of red pigments using ultrasonic-assisted extraction (18.18 ± 0.08% and 13.11 ± 0.06%), instead of overnight extraction (11.86 ± 0.05% and 11.10 ± 0.08%). In principle, carotenoids and anthocyanins are responsible for the red color of plant extracts [73]. The main anthocyanin in red grapes is malvidin-3-glycoside [64], while different anthocyanins have been found in red onion skins, such as 3-(6”-malonylglucoside) and cyanidin 3-(6-malonylglucoside), with the cyanidin 3-O-glucoside being the primary anthocyanin in the epidermal cells of red onions [45,46].

### 2.6. pH and Total Soluble Solids

The representative pH and total soluble solids of OPP, MWP, and SWP aqueous extracts are shown in Table 4. Generally, pH is one of the most important factors in food safety, as a reduced pH inhibits bacterial growth [74]. MWP recorded the lower pH value, at 5.18 ± 0.01, followed by SWP at 5.21 ± 0.02 and OPP at 5.30 ± 0.01, with statistically significant (*p* < 0.05) differences. In general, the skin of red onions has a slightly acidic pH, primarily attributed to the phenolic acids it contains, such as ferulic acid, gallic acid, protocatechuic acid, vanillic acid, and coumaric acid [13]. Bedrníček et al. [74] previously recorded an acidic pH value in OPP (4.65 ± 0.06), supporting that hypothesis. Considering the grape pomace extracts, they usually show pH values ranging from 3.4 to 5.4, while Rodrigues et al. [51] recorded a pH value of 3.88 in grape pomace extracts. Also, Deng et al. [75] obtained a pH value of 3.65 for Merlot pomace extract with preheated water. The acidic pH of grape pomace is attributed to the presence of phenolic compounds and fatty acids [26,27,29,30,51].

OPP exhibited the highest TSS value with 1.14 ± 0.05 °Brix, followed by MWP with 0.84 ± 0.05 °Brix and SWP with 0.71 ± 0.08 °Brix, with statistically significant (*p* < 0.05) differences. Onions are a rich source of polysaccharides [13], and their content varies depending on the variety [76]. Sarkar et al. [77] and Poursakhi et al. [78] recorded TSS values ranging from 4 to 9 °Brix in different raw bulb varieties. Also, grape pomace contains unfermented sugars that may be left in the pomace during the fermentation process, whereas their concentration depends on the fermentation stage of the wine. Previously, Deng et al. [75] reported a TSS value of 3.60 ± 0.00 °Brix in Merlot grape pomace. Moreover, in the study by Bredun et al. [79], a value of 0.9 ± 0.1 °Brix was recorded in grape pomace, strongly supporting our previous hypothesis.

### 2.7. Characterization of Ethanol of Grape Origin (EEGO) with UV-Vis and ATR-FTIR Spectra

**Figure 1:**
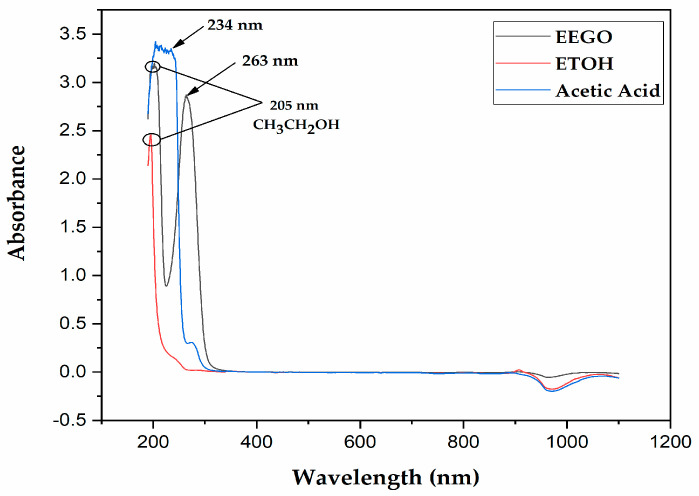
UV-Vis spectra of ethanol of grape origin (EEGO), absolute ethanol (CH_3_CH_2_OH, ETOH), and acetic acid.

The UV-Vis spectra of EEGO, absolute ethanol, and acetic acid are represented in Figure 1. There was an effort to monitor if any peaks were obtained in the ultraviolet-visible region of EEGO, and indeed, two sharp peaks were identified at 205 nm and 263 nm. After a full scan of absolute ethanol (as a standard), a peak was also identified at 205 nm, confirming that this peak corresponds to the presence of ethanol in EEGO. Moreover, standard acetic acid showed a peak at 234 nm after the full range scan. In our opinion, due to matrix effects in the EEGO sample, the acetic acid peak was slightly moved to the right.

**Figure 2:**
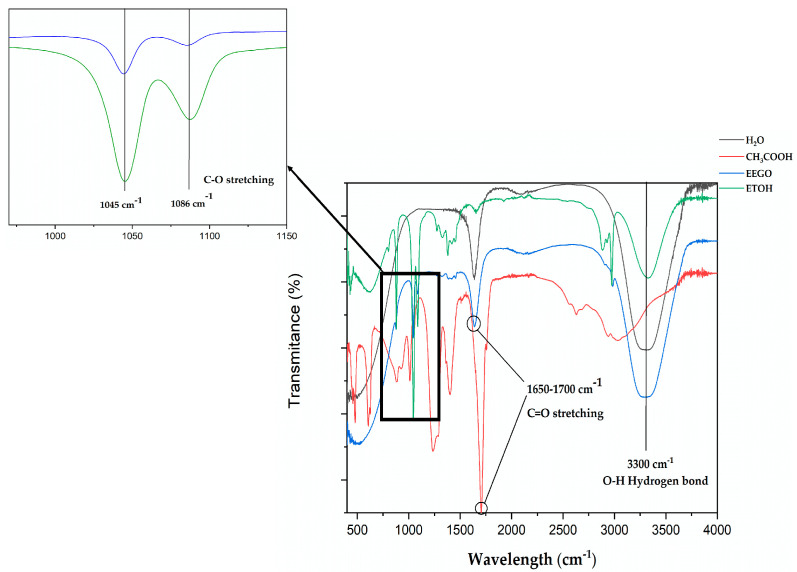
ATR-FTIR spectra of ethanol of grape origin (EEGO), acetic acid, distilled water, and absolute ethanol (ETOH).

The ATR-FTIR spectra of EEGO, distilled water, absolute ethanol, and acetic are represented in Figure 2. The intense band of EEGO at 3300 cm^−1^ is attributed to the O-H stretching and O-H-O scissors bending of water [80]. This could be claimed by comparing it with the pure water spectra. Furthermore, a sharp peak in EEGO was identified at 1650 cm^−1^. This sharp peak in the 1650–1700 cm^−1^ region corresponds to the C=O stretching of carboxylic acids [81]. In comparison with the standard acetic acid, it can be seen that it shows a strong and sharp peak at 1700 cm^−1^. This slight movement is probably due to matrix effects in the EEGO sample, as we mentioned earlier. Finally, a sharp peak was identified at 1045 cm^−1^ in the EEGO sample, followed by a smaller one at 1086 cm^−1^. Comparing these peaks in that area with the spectra of absolute ethanol, it can be clearly seen that they have the same shape. Also, these peaks at 1045 cm^−1^ and 1086 cm^−1^ correspond to the C-O bond of ethyl alcohol, as Mudalip et al. [82] recorded the same shape at 1048 cm^−1^ and 1092 cm^−1^, respectively.

## 3. Materials and Methods

### 3.1. Preparation of Onion Peel and Grape Pomace Powder

Approximately 4 kg of red skin onions cultivated in the village of Paravola (Agrinio, Aitoloakarnania, Greece) were purchased from a local greengrocer and brought to the laboratory. The onions were washed with tap water and dried using a paper towel before being cut in half with a knife, and the first two layers were removed to obtain the onion peels. Afterwards, the peels were placed in a laboratory oven (Memmert Universal Oven UM 400, Leverkusen, Germany) at 50 ± 1 °C for 48 h to dry. Similarly, pomace from the Merlot grape variety was transferred from Kourtis Winery (Stimagka, Korinthia, Greece) two days after fermentation was completed (17 October 2024). Additionally, pomace from the Syrah grape variety was donated by Petrino Horio Winery (Thermo, Aitoloakarnania, Greece) on the same day that fermentation was completed (24 September 2024). The grape pomace from Merlot and Syrah grape varieties were dried in the oven mentioned above, at 48 ± 1 °C for 36 h. Then, the dried onion peels, Merlot, and Syrah grape pomace were pulverized using a blender (R-583, Rohnson, Prague, Czech Republic) to obtain a fine powder with a pore size of approximately 40–50 μm. Finally, onion peel powder (OPP), Merlot winery powder (MWP), and Syrah winery powder (SWP) were stored at −18 ± 2 °C and utilized until the experiments were completed.

### 3.2. Chemicals and Reagents

Sodium carbonate (Na_2_CO_3_), sodium nitrite (NaNO_2_) and acetate buffer (CH_3_COONa × 3H_2_O) were purchased from Penta (Prague, Czech Republic). Folin–Ciocalteu and aluminum chloride (AlCl_3_) were purchased from Sigma-Aldrich (Darmstadt, Germany). 2,2-Diphenyl-1-picrylhydrazyl (DPPH) was purchased from TCI (Tokyo, Japan) and absolute ethanol (CH_3_CH_2_OH), pure methanol (CH_3_OH) and potassium chloride (KCl) were purchased from Merck (Darmstadt, Germany). 2,4,6-Tris(2-pyridil)-s-triazine (TPTZ) was purchased from Glentham Life Sciences (Corsham, UK). 6-hydroxy-2,5,7,8-tetramethylchroman-2-carboxylic acid (Trolox) was purchased from BLD Pharm (Shangai, China). FeCl_3_ × 6H_2_O was purchased from Honeywell (Charlotte, NC, USA). Gallic acid (3,4,5-trihydrobenzoic acid) 99% isolated from *Rhus chinensis* Mill. was purchased from JNK Tech. Co. (Seongnam, Republic of Korea). Sodium hydroxide (NaOH) was purchased from Lach-Ner (Zagreb, Croatia). Acetic acid glacial was purchased from Chem-Lab (Zedelgem, Belgium).

### 3.3. Preparation of Ethanol of Grape Origin

The first of the two different solvents was prepared following the simple distillation method introduced by Lazaridis et al. [62]. In brief, 300 mL of dry white wine was distilled until 200 mL of ethanol (14% Vol.) was collected. This ethanol is of grape origin and comprises an eco-friendly and bi-phase solvent (aqueous-organic) to extract phytochemicals from various foods.

### 3.4. Extraction of Phenolic Compounds

The phenolic compounds of OPP, MWP, and SWP were extracted using two different solvents: (i) ethanol of grape origin and (ii) ethanol/water 70/30. Firstly, 1 g of each powder was placed in a Falcon tube, and 15 mL of each solvent was added. Then, two different extraction methods after optimization were carried out: (i) overnight extraction at room temperature (25 °C) and (ii) ultrasonic-assisted extraction (ZCC017, Nahita, Auxilab, Navarra, Spain), for 15 min at room temperature, with ultrasonic frequency of 40KHz and ultrasonic power of 120 W, to compare which one achieves the best recovery of phenolic compounds. Finally, the samples were filtered using a 0.45 μm Whatman filter, and the extracts were kept under refrigeration (4 ± 2 °C) until the end of the experiments. The selected extraction conditions were based on the optimal conditions summarized in the study of Kumar et al. [83].

### 3.5. Determination of Total Phenolic Content

The total phenolic content of the extracts was determined using the Folin–Ciocalteu method described analytically by Kitsios et al. [84]. Specifically, 2.5 mL of deionized water was transferred to a volumetric flask, followed by 200 μL of each extract and 250 μL of the Folin–Ciocalteu reagent. The mixture was left for 3 min, and then 0.5 mL of saturated sodium carbonate (Na_2_CO_3_, 30% *w/v*) was added. The solution was then brought to a final volume of 5 mL with deionized water. The solution was left in the dark at room temperature for 2 h before the absorbance was measured at λ = 760 nm using a UV-Vis spectrophotometer (UV-1280, Shimadzu, Kyoto, Japan). Using gallic acid as a standard, a calibration curve was prepared (0–500 ppm), plotting the gallic acid concentration (x) versus absorbance (y) as following Equation (1):

y = 0.0044x + 0.0082; *R*^2^ = 0.9982 (1)

Results were expressed as milligrams of gallic acid equivalents (GAE) per gram of dry extract (mg GAE/g) out of five replicates (n = 5).

### 3.6. Determination of Total Flavonoid Content

#### 3.6.1. Method Development

**Figure 3:**
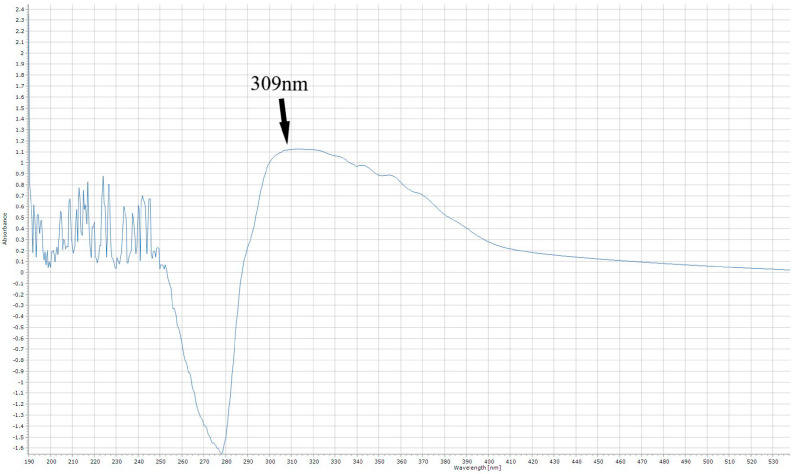
The maximum absorption of quercetin, after the aluminum chloride reaction.

The total flavonoid content of the extracts was determined using a method developed in our laboratory. Before developing our method, we considered some relevant studies in the literature that reported the maximum absorption spectra of quercetin [85,86]. Firstly, a quercetin standard solution of 300 ppm was prepared by dissolving 0.0075 g of quercetin in 25 mL of absolute ethanol. Then, 250 μL of the quercetin standard solution was transferred to a glass test tube, followed by 150 μL of 5% NaNO_2_ (*w/v*), and the mixture was left in the dark for 5 min. Afterwards, 150 μL of 10% (*w/v*) AlCl_3_ was added to the mixture, followed by 1 mL of 1 M NaOH and 5 mL of deionized water. The mixture was allowed to incubate for 30 min in the dark at room temperature, and then 3 mL was transferred to a Quartz cuvette. The cuvette was placed in the spectrophotometer mentioned above and was adjusted for 1 nm spectral analysis at the scan range 1100–190 nm. We observed that the peak detection of quercetin was at 309 nm, as represented in Figure 3.

#### 3.6.2. Preparation of Quercetin Calibration Curve

A quercetin standard solution (500 ppm) was prepared by adding 0.0125 g of quercetin and 25 mL of absolute ethanol in a volumetric flask. After proper dilutions, 100, 200, 300, and 400 ppm solutions were prepared. The calibration curve was plotted as concentration (x) versus absorbance (y) as following Equation (2):

y = 0.0026x + 0.2897; *R*^2^ = 0.9938 (2)

#### 3.6.3. Determination of Flavonoids in OPP, MWP, and SWP Extracts

The total flavonoid content of the extracts was determined using the reaction method mentioned in Section 3.6.1. After the measurements were completed, the concentration was calculated using Equation (2). Each sample was analyzed in quintuplicate (n = 5), and results were expressed as milligrams of quercetin equivalents (QE) per gram of dry extract (mg QE/g).

### 3.7. Total Anthocyanin Content

The anthocyanin content of the extracts was determined using the differential pH method, according to Wrolstad [87]. That method is based on the reversible color change produced by anthocyanins when the pH changes. Firstly, each extract was diluted 1:5 (*v/v*) with 0.025 M of potassium chloride (pH = 1.0) and 0.4 M of sodium acetate (pH = 4.5). The anthocyanin content was calculated using Equation (3):

(3) $$TAC=\frac{A\times MW\times DF\times 1000}{\varepsilon \times l}$$

where the absorption of the sample A = (A510_nm_ − A700_nm_) pH 1.0 − (A510_nm_ − A700_nm_) pH 4.5, MW is the molecular weight of cyanidin-3-glycoside (C3G) (449.2), DF is the dilution factor used (DF = 1), ε is the molar absorption of C3G, and l is the path length of the cuvette (1 cm). Each sample was measured in quintuplicate (n = 5) and results were expressed as milligrams of C3G per gram of dry extract (mg C3G/g).

### 3.8. Determination of In Vitro Antioxidant Activity in OPP, MWP, and SWP Extracts Using DPPH Assay

#### 3.8.1. Preparation of DPPH^.^ Standard Solution

Firstly, a standard DPPH^.^ solution (1.01 × 10^−4^ M) was prepared by dissolving 0.01 g of DPPH^.^ in 250 mL of absolute ethanol, under stirring. To ensure its neutrality, the solution pH was measured (7.02 ± 0.01), using a portable pH meter (pHEP+, HANNA Instruments, HI9808, Athens, Greece). Then, the solution was covered with aluminum foil and left for 2 h in 4 ± 1 °C to stabilize before the experiments took place.

#### 3.8.2. Preparation of DPPH^.^ Calibration Curve

A calibration curve was prepared after proper dilutions of the standard DPPH^.^ solution. The obtained solutions (at least 5 replicates) were vortexed and their absorbance was measured at λ=517 nm using the spectrophotometer mentioned previously. The calibration curve of concentration (x) versus absorbance (y) was expressed by the following Equation (4):

y = 0.0319x + 0.0026; *R*^2^ = 0.9997 (4)

#### 3.8.3. Estimation of Antioxidant Activity in Food Waste By-Products Using the DPPH Assay

The antioxidant activity of the polyphenolic extracts was determined following the DPPH^.^ reaction method of Lazaridis et al. [88]. Briefly, 100 μL of each extract was placed in a cuvette, followed by 1 mL of acetate buffer (0.1 M) and 1.9 mL of DPPH^.^ solution. The mixture was left in the dark, and the absorbance was measured at λmax = 517 nm, until the reaction reached a plateau. The antioxidant activity of each sample was calculated using the following Equation (5):

AA (%) = (A_0_ − A_t_/(A_0_) × 100 (5)

where A_0_ is the initial absorbance of DPPH^.^ solution and A_t_ is the absorbance of the remaining DPPH, after its reaction with the phenolic compounds of the extracts. Each sample was analyzed in quintuplicate (n = 5).

#### 3.8.4. Preparation of Trolox Calibration Curve Against DPPH

Briefly, 25 mg of Trolox were transferred to a volumetric flask and dissolved in 10 mL of pure methanol. Then, 90 mL of distilled water was added to a final volume of 100 mL and obtain a concentration of 1000 μM. After initial dilutions to proper concentrations, the standard solutions were tested against DPPH, and a calibration curve using the AA% (y) against the concentration (x) was prepared between 200 and 1000 μM, as shown in Equation (6):

y = 0.0318x + 9.7931; *R*^2^ = 0.9996 (6)

Each sample was analyzed in quintuplicate, and results were expressed as μmol Trolox equivalents/L of extract.

### 3.9. Determination of In Vitro Antioxidant Activity in OPP, MWP, and SWP Extracts Using the FRAP Assay

#### 3.9.1. Preparation of Ferric Reducing Antioxidant Power (FRAP) Reagent

The FRAP reagent was freshly prepared as described by Karabagias et al. [89]. 2.5 mL of 5 mM TPTZ solution was transferred to 40 mM HCl, followed by 2.5 mL of 20 mM FeCl_3_ × 6H_2_O and 25 mL of acetate buffer (pH = 3.6). The final solution was kept in the dark until the reaction.

#### 3.9.2. Determination of Antioxidant Activity in OPP, MWP, and SWP Extracts Using FRAP Assay

The FRAP assay was performed using the method of Benzie and Strain [90] with some modifications. Briefly, 0.2 mL of each extract was mixed with 1.8 mL of pre-warmed FRAP solution at 37 °C. The reaction mixture was left in the dark at 37 °C for 10 min, and the absorbance was measured at λ = 593 nm. Each sample was analyzed in quintuplicate (n = 5), and the results were expressed as mmol of Fe^2+^/L extract using the calibration curve represented in the study of Karabagias et al. [89].

#### 3.9.3. Preparation of Trolox Calibration Curve Against FRAP

The Trolox equivalents calibration curve was prepared using the aforementioned Trolox solutions in different concentrations (x), against the obtained absorbance (y), after their reaction with the FRAP reagent. The obtained standard curve between 20 and 500 μM is shown in Equation (7):

y = 0.0034x + 0.4186; *R*^2^ = 0.9945 (7)

Each sample was analyzed in quintuplicate (n = 5) and results were expressed as μmol Trolox equivalents/L extract.

### 3.10. Determination of Color Tones

The color parameters of OPP, MWP and SWP extracts were determined basis on the optical density (OD) measurements at λ = 420 nm (YCT, Yellow color tone), λ = 520 nm (BCT, Blue color tone) and λ = 620 nm (RCT, Red color tone) (using the aforementioned spectrophotometer) according to Lazaridis et al. [62]. The color parameters were calculated as follows in Equations (8)–(10):

YCT (%) = (A420/CI) × 100(8)

BCT (%) = (A520/CI) × 100(9)

RCT (%) = (A620/CI) × 100(10)

where CI: Color intensity A420 + A520 + A620. Color tones were expressed as % values out of five replicates (n = 5).

### 3.11. pH and Total Soluble Solids (TSS)

Before the effective acidity (pH) and TSS measurements, 10 g of each powder was placed in a beaker and dissolved in 100 mL of deionized water (10% *w/v*). The mixture was vortexed for 5 min and finally filtered using a filter paper. The filtered extract was used for the pH and TSS measurements. pH measurements were performed by placing 20 mL of each extract in a beaker. Then, a portable pH meter (pHEP+, HANNA Instruments, HI9808, Athens, Greece) was immersed in the extract. The results were expressed as pH units (20 °C) and originated from five replicates (n = 5). TSS measurements were determined using a portable refractometer (RB 32 ATC, Hanna Instruments, Greece), after transferring 100 μL of the aqueous extract onto the prism of the refractometer. Results were expressed as °Brix values and originated from five replicates (n = 5).

### 3.12. Characterization of Ethanol of Grape Origin (EEGO) with Ultraviolet–Visible and Attenuated Total Reflectance–Fourier Transform Infrared (ATR-FTIR) Spectroscopy

UV-Vis spectra were recorded using the spectrophotometer mentioned previously. Briefly, 3 mL of ethanol of grape origin, acetic acid, and absolute ethanol, respectively, were transferred to a Quartz cuvette and subjected to a full scan in the range of 1100–190 nm. A baseline correction was performed using distilled water. FTIR spectra of ethanol of grape origin, absolute ethanol, acetic acid, and distilled water were obtained using a Shimadzu IRSpirit-TX spectrophotometer (Shimadzu, Kyoto, Japan). Approximately 100 μL of each sample was transferred to the ATR receptor, and the spectra were recorded over the wavenumber 4000–400 cm^−1^, after 64 scans and at a resolution of 4 cm^−1^.

### 3.13. Statistical Analysis

The research data were subjected to one-way analysis of variance (ANOVA) to monitor any statistically significant (*p <* 0.05) differences between the average values of the determined parameters with the different methods of phenolic compounds extraction, concerning each case (OPP, MWP, and SWP). The method of extraction (and/or the type of food waste by-product) was considered as the group factor variable, whereas the determined parameters were considered as the independent variable. Multiple comparison test (post hoc analysis) was also carried out to indicate any statistically significant (*p* < 0.05) differences between pairs of phenolic compounds’ extraction methods, according to Tukey’s honestly significant difference (HSD) test. Statistical analysis was performed using the Statistical Package for the Social Sciences (SPSS) statistics software, version 28.0 (SPSS, IBM Inc., Armonk, NY, USA, 2021).

## 4. Conclusions and Future Perspectives

Food waste by-products contain a high number of bioactive compounds that possess high antioxidant activity, including phenolic compounds, resulting in various biochemical and physicochemical properties. The results of the present study showed that the solvent and the extraction method (overnight or ultrasonic-assisted extraction) that were used for the recovery of these bioactive compounds play a significant role in the type of bioactive compound that is aimed at being determined/utilized (i.e., total phenolic compounds, anthocyanins, flavonoids, etc.), along with their recovery yield. In this context, we should emphasize the use of ethanol of grape origin as a solvent for the recovery of bioactive components. A solvent that is characterized by the presence of grape-derived co-solvents such as acetic acid, which resulted in the high recovery of anthocyanins during the ultrasonic-assisted extraction of winery pomace. In addition, compared to conventional or other green emerging solvents (i.e., eutectic solvents), ethanol of grape origin might have potential advantages (e.g., sustainability, scalability, cost reduction, etc.), and thus, a possible impact on economic and industrial factors. Overall, the high concentration of phenolic compounds in agricultural food waste by-products and their extraction with a grape-derived solvent is a useful tool to develop in the future, food and animal feed supplements, or packaging materials with antibacterial and antioxidant properties for the shelf-life evaluation of foods of animal and plant origin.

## Figures and Tables

**Table 1:** Total phenolic content, total flavonoid content, and total anthocyanin content of onion peel and grape pomace extracts during different extraction methods.

Food Waste Powder	Extraction	TPC (mg GAE/g)	TFC (mg QE/g)	TAC (mg C3G/g) *
OPP	EEGO Overnight	16.00 ± 0.31 ^a^	2.01 ± 0.02 ^a^	0.10 ± 0.00
OPP	EEGO UAE	16.59 ± 0.96 ^a^	2.23 ± 0.28 ^a^	0.08 ± 0.00
OPP	70% Ethanol Overnight	16.15 ± 0.77 ^a^	3.66 ± 0.07 ^b^	-
OPP	70% Ethanol UAE	15.58 ± 0.43 ^a^	3.14 ± 0.19 ^c^	-
MWP	EEGO Overnight	9.43 ± 0.19 ^a^	1.91 ± 0.06 ^a^	0.45 ± 0.00
MWP	EEGO UAE	8.61 ± 0.12 ^a^	1.99 ± 0.36 ^a^	0.62 ± 0.00
MWP	70% Ethanol Overnight	11.62 ± 1.91 ^b^	5.63 ± 0.03 ^b^	-
MWP	70% Ethanol UAE	16.40 ± 0.93 ^c^	5.52 ± 0.27 ^b^	-
SWP	EEGO Overnight	11.12 ± 0.20 ^a^	1.99 ± 0.16 ^a^	0.28 ± 0.00
SWP	EEGO UAE	9.93 ± 0.26 ^a^	2.73 ± 0.20 ^b^	0.28 ± 0.00
SWP	70% Ethanol Overnight	14.43 ± 1.46 ^b^	6.28 ± 0.62 ^c^	-
SWP	70% Ethanol UAE	15.68 ± 0.96 ^b^	4.71 ± 0.22 ^d^	-

OPP: Onion peel powder. MWP: Merlot winery pomace powder. SWP: Syrah winery grape pomace. EEGO: Ethanolic extract of grape origin. UAE: Ultrasonic-assisted extraction. TPC: total phenolic content expressed as gallic acid equivalents (GAEs) per gram of dry extract. TFC: total flavonoid content expressed as quercetin equivalents (QE) per gram of dry extract. TAC: total anthocyanin content expressed as milligrams of cyanidin-3-glycoside (C3G) per gram of dry extract. Different letters in each column (^a–d^) indicate statistically significant (*p* < 0.05) differences according to Tukey’s honestly significant difference (HSD) test, between the same powder subjected to different extraction methods. * Tukey’s HSD test cannot be computed, given the existence of only two determinations in each food waste powder (minimum demand of 3 determinations for the SPSS program).

**Table 2:** Antioxidant activity of onion peel and grape pomace extracts during different extraction methods.

Food Waste Powder	Extraction	DPPH (%)	DPPH (μmol TE/L)	FRAP (mmol Fe^2+^/L)	FRAP (μmol TE/L)
OPP	EEGO Overnight	63.70 ± 1.43 ^a^	1695.19 ± 45.10 ^a^	324.57 ± 2.00 ^a^	1102.59 ± 8.23 ^a^
OPP	EEGO UAE	62.60 ± 0.69 ^a^	1660.58 ± 21.72 ^a^	328.14 ± 4.00 ^a^	1117.29 ± 16.47 ^a^
OPP	70% Ethanol Overnight	53.70 ± 2.18 ^b^	1380.73 ± 68.78 ^b^	336 ± 4.14 ^b^	1149.65 ± 17.06 ^b^
OPP	70% Ethanol UAE	54.82 ± 1.38 ^b^	1.416.08 ± 43.29 ^b^	331.64 ± 0.65 ^ab^	1131.71 ± 2.64 ^ab^
MWP	EEGO Overnight	71.17 ± 0.34 ^a^	1930.05 ± 10.55 ^a^	318.14 ± 3.43 ^a^	1076.12 ± 14.12 ^a^
MWP	EEGO UAE	67.86 ± 1.73 ^b^	1825.96 ± 54.33 ^b^	323.14 ± 4.14 ^a^	1096.71 ± 17.06 ^a^
MWP	70% Ethanol Overnight	52.94 ± 1.66 ^c^	1356.86 ± 52.35 ^c^	339.50 ± 1.79 ^b^	1164.06 ± 7.35 ^b^
MWP	70% Ethanol UAE	53.29 ± 0.94 ^c^	1367.71 ± 29.73 ^c^	345.79 ± 2.50 ^c^	1189.94 ± 10.29 ^c^
SWP	EEGO Overnight	68.82 ± 2.55 ^a^	1856.06 ± 80.23 ^a^	327.29 ± 2.28 ^a^	1113.76 ± 9.41 ^a^
SWP	EEGO UAE	68.85 ± 2.40 ^a^	1857.04 ± 75.36 ^a^	336.36 ± 1.78 ^a^	1151.12 ± 7.35 ^a^
SWP	70% Ethanol Overnight	50.56 ± 1.50 ^b^	1281.88 ± 47.04 ^b^	357.07 ± 9.36 ^b^	1236.41 ± 38.53 ^b^
SWP	70% Ethanol UAE	46.23 ± 3.39 ^b^	1145.74 ± 106.74 ^b^	360.14 ± 4.71 ^b^	1249.06 ± 19.41 ^b^

EEGO: Ethanolic extract of grape origin, UAE: Ultrasonic-assisted extraction. FRAP: Ferric-reducing antioxidant power. TE: Trolox equivalents. Different letters in each column (^a–c^) indicate statistically significant (*p* < 0.05) differences according to Tukey’s honestly significant difference (HSD) test, between the same powder subjected to different extraction methods.

**Table 3:** Color parameters of onion peel and grape pomace extracts during different extraction methods.

Food Waste Powder	Extraction	YCT (%)	BCT (%)	RCT (%)
OPP	EEGO Overnight	77.99 ± 0.26 ^a^	17.94 ± 0.18 ^a^	4.19 ± 0.07 ^a^
OPP	EEGO UAE	79.78 ± 0.09 ^b^	17.03 ± 0.07 ^b^	3.09 ± 0.04 ^b^
OPP	70% Ethanol Overnight	78.55 ± 0.07 ^c^	19.13 ± 0.09 ^c^	4.00 ± 0.07 ^c^
OPP	70% Ethanol UAE	76.25 ± 0.08 ^d^	18.43 ± 0.06 ^d^	5.10 ± 0.04 ^d^
MWP	EEGO Overnight	37.43 ± 0.02 ^a^	48.18 ± 0.01 ^a^	14.39 ± 0.02 ^a^
MWP	EEGO UAE	39.94 ± 0.01 ^b^	43.13 ± 0.01 ^b^	16.92 ± 0.01 ^b^
MWP	70% Ethanol Overnight	45.72 ± 0.16 ^c^	40.13 ± 0.17 ^c^	14.14 ± 0.11 ^c^
MWP	70% Ethanol UAE	45.84 ± 0.38 ^c^	40.00 ± 0.36 ^c^	14.16 ± 0.18 ^c^
SWP	EEGO Overnight	42.57 ± 0.09 ^a^	45.57 ± 0.11 ^a^	11.86 ± 0.05 ^a^
SWP	EEGO UAE	43.65 ± 0.10 ^b^	38.17 ± 0.11 ^b^	18.18 ± 0.08 ^b^
SWP	70% Ethanol Overnight	53.22 ± 0.08 ^c^	35.68 ± 0.07 ^c^	11.10 ± 0.08 ^c^
SWP	70% Ethanol UAE	51.90 ± 0.24 ^d^	34.99 ± 0.22 ^d^	13.11 ± 0.06 ^d^

YCT: Yellow color tone, BCT: Blue color tone, RCT: Red color tone. Different letters in each column (^a–d^) indicate statistically significant (*p* < 0.05) differences according to Tukey’s honestly significant difference (HSD) test, between the same powder subjected to different extraction methods.

**Table 4:** Representative pH and TSS values of OPP, MWP, and SWP aqueous extracts.

Food Waste Powder	pH	TSS (°Brix)
OPP	5.30 ± 0.01 ^a^	1.14 ± 0.05 ^a^
MWP	5.18 ± 0.01 ^b^	0.84 ± 0.05 ^b^
SWP	5.21 ± 0.02 ^c^	0.71 ± 0.08 ^c^

Different letters in each column (^a–c^) indicate statistically significant (*p* < 0.05) differences between the food waste powders according to Tukey’s honestly significant difference (HSD) test.

## Data Availability

The original contributions presented in this study are included in the article. Further inquiries can be directed to the corresponding author.
